# The real culprit behind diabetic nephropathy: impaired renal autoregulation?

**DOI:** 10.14814/phy2.13138

**Published:** 2017-03-14

**Authors:** Ruisheng Liu

**Affiliations:** ^1^ Department of Molecular Pharmacology & PhysiologyUniversity of South Florida College of MedicineTampaFlorida

More than 29 million of Americans have diabetes. About 30–40% of the diabetic patients will eventually develop diabetic nephropathy (DN), which is the leading cause of end‐stage renal disease (Centers for Disease Control and Prevention, 2014; United States Renal Data System., 2012). Control of blood pressure (BP) and blood glucose has been shown to reduce the risk of developing DN (Nathan 2014). However, not all patients with poor control in BP and plasma glucose will develop renal disease. On the other hand, over 1/3 of patients with type 2 diabetes develop DN despite strict control of BP and/or blood glucose (Mooyaart 2014; Tomino and Gohda 2015). Therefore, further understanding the pathophysiological mechanisms underlying the DN is essential to identify potential target for treatment.

Many studies demonstrated an association between glomerular capillary hypertension and diabetes‐associated kidney disease. An elevated glomerular capillary pressure was first reported in experimental diabetic Munich‐Wistar rats in 1980s (Hostetter et al. 1982). In physiological conditions, glomerular capillary pressure is well‐maintained within a normal range by a renal autoregulatory mechanism, by which the renal blood flow (RBF) and glomerular filtration rate (GFR) maintain constant and are independent of systemic blood pressure over a wide range. While its mechanism and significance have not been fully elucidated, renal autoregulation is considered an essential mechanism to keep constant electrolytes delivery to distal nephrons and sustain normal kidney clearance function facing fluctuations in systemic blood pressure. Renal autoregulation is also proposed to be a unique self‐protective mechanism of the kidney in our body, which isolate fluctuations in systemic blood pressure and keep a relatively constant level in intraglomerular capillary pressure that may prevent glomerular injuries. The renal autoregulation is believed to be mainly mediated by myogenic response of the afferent arteriole and tubuloglomerular feedback (TGF) response. Myogenic response is rapid response of vasoconstriction of the afferent arteriole in response to an increase in transmural pressure. TGF response refers a negative feedback mechanism that increased NaCl delivery to the macula densa promotes release of adenosine and/or ATP, which constricts the afferent arteriole and decreases single nephron GFR (Burke et al. 2014).

Impaired autoregulation has been proposed to play an important role in the development of kidney injury by elevation of glomerular capillary pressure in hypertensive experimental models. Typical examples are Dahl salt‐sensitive rats and spontaneously hypertensive (SHR) rats. Dahl salt‐sensitive rats exhibit impaired autoregulation and are sensitive to diabetic kidney injury, whereas SHR rats show intact autoregulation and are resistant to diabetic kidney injury in the present of hypertension (Slaughter et al. 2013). However, whether the impaired autoregulation itself without hypertension induces diabetic kidney injury remains elusive. It has long been reported that Milan normotensive strain (MNS) of rats develops progressive proteinuria and glomerular injury following the induction of diabetes with streptozotocin (STZ) (Pugliese et al. 2005) in the absence of systemic hypertension. In contract, Milan hypertensive strain (MHS) of rats is resistant to the development of proteinuria and kidney injury. The mechanisms underlying have not been clarified.

A recent study by Ge et al. (2016) provided a potential mechanism for the difference in diabetes‐associated kidney injury between MNS and MHS rats. They demonstrated that normotensive rats with impaired autoregulation developed severe diabetic kidney injury, in contrast, the hypertensive rats with intact autoregulation were protected from diabetic kidney injury. Renal autoregulation was evaluated by measurement of myogenic response of the afferent arteriole (Af‐Art), RBF and stop‐flow pressure of proximal tubule when perfusion pressure was increased in MNS and MHS rats. While MHS exhibited efficient renal autoregulatory function, autoregulation in MNS was impaired. Then type 1 diabetes was induced with STZ and plasma glucose levels were maintained at a similar level in both groups of animals. After 21 weeks of induction of diabetes, MAP was over 20 mmHg higher in MHS than MNS rats. The MNS rats developed severe diabetic kidney injury reflected by proteinuria, plasma creatinine, and renal histology. However, the MHS rats were largely protected from renal injury. This study highlighted the essential role of renal autoregulation in the development of diabetic kidney injury independent of hypertension. Unlike in Munich‐Wistar rats, glomerular capillary pressure is difficult to be measured directly in most other strains of rodents with micropuncture since the glomeruli are not visible on the kidney surface. Thereby, stop‐flow pressure was used as a surrogate for glomerular capillary pressure in this study. It should be aware that the using stop‐flow pressure for evaluation of autoregulation excludes the contribution of TGF response. Thereby the impairment of autoregulation could be underestimated if both myogenic response and TGF response are impaired in MNS. Actually, the results of RBF implied this possibility. RBF raised about 40%, whereas stop‐flow pressure only elevated by about 20% when renal perfusion pressure increased from 100 to 140 mmHg (Ge et al. 2016).

Considering still no efficient treatment for DN exist, therefore, we are speculating that in the prevention and treatment of diabetic nephropathy we might have been shooting the wrong targets by focusing on accomplices such as hypertension, hyperglycemia, dyslipidemia, obesity, and insulin resistance, but missed the real culprit – the impaired renal autoregulation. Candidates for regulation of renal autoregulation include but are not limited to Adducin‐3 gene (Burke et al. 2013) and TRP channels (Carlstrom et al. 2015) for myogenic response of the Af‐Art and NOS1 splice variants in the macula densa for TGF responsiveness (Lu et al. 2010, 2016). These candidates may be regulated by both genetic and environmental factors and could be potential targets for the prevention and treatment for DN.
